# Dominant optic atrophy, OPA1, and mitochondrial quality control: understanding mitochondrial network dynamics

**DOI:** 10.1186/1750-1326-8-32

**Published:** 2013-09-25

**Authors:** Marcel V Alavi, Nico Fuhrmann

**Affiliations:** 1 Department of Ophthalmology, University of California, San Francisco, 10 Koret Way, 94143-0730 San Francisco, CA, USA; 2 Institut für Medizinische Genetik und Molekulare Medizin, Köln, Germany

**Keywords:** DOA, LHON, Glaucoma, OPA1, OPA3, BNIP3, NMDA receptors, Oxidative stress, Mitochondrial fusion, Retinal ganglion cells, Glutamate excitotoxicity, Mitochondrial quality control, Mitochondrial optic neuropathies

## Abstract

Mitochondrial quality control is fundamental to all neurodegenerative diseases, including the most prominent ones, Alzheimer’s Disease and Parkinsonism. It is accomplished by mitochondrial network dynamics – continuous fission and fusion of mitochondria. Mitochondrial fission is facilitated by DRP1, while MFN1 and MFN2 on the mitochondrial outer membrane and OPA1 on the mitochondrial inner membrane are essential for mitochondrial fusion. Mitochondrial network dynamics are regulated in highly sophisticated ways by various different posttranslational modifications, such as phosphorylation, ubiquitination, and proteolytic processing of their key-proteins. By this, mitochondria process a wide range of different intracellular and extracellular parameters in order to adapt mitochondrial function to actual energetic and metabolic demands of the host cell, attenuate mitochondrial damage, recycle dysfunctional mitochondria via the mitochondrial autophagy pathway, or arrange for the recycling of the complete host cell by apoptosis. Most of the genes coding for proteins involved in this process have been associated with neurodegenerative diseases. Mutations in one of these genes are associated with a neurodegenerative disease that originally was described to affect retinal ganglion cells only. Since more and more evidence shows that other cell types are affected as well, we would like to discuss the pathology of dominant optic atrophy, which is caused by heterozygous sequence variants in *OPA1*, in the light of the current view on OPA1 protein function in mitochondrial quality control, in particular on its function in mitochondrial fusion and cytochrome C release. We think OPA1 is a good example to understand the molecular basis for mitochondrial network dynamics.

## Introduction

Mitochondrial dynamics become more and more important since it is recognized that the morphology of this highly dynamic network is relevant for many pathological conditions, foremost neurodegeneration [1] but also stroke [2] and cancer [3,4]. Mitochondrial network dynamics – continuous fission and fusion of mitochondria – mediates mitochondrial quality control (MQC) for the eukaryotic cell. MQC comprises more than one would initially associate with the term keeping mitochondria in “good health” by restoring or removing damaged organelles. But mitochondria are capable to process a wide range of different intracellular and extracellular parameters by mitochondrial network dynamics and they do this to accommodate cell homeostasis and cell fate by the following measures: a) Mitochondrial network morphology changes to either a more filamentous or more fragmented state to adapt mitochondrial function to actual energetic and metabolic demands of the host cell [5,6]. b) Fusion of dysfunctional mitochondria to functional mitochondria can attenuate damage to mitochondrial proteins, lipids, and mtDNA [7]. c) Inhibition of mitochondrial fusion targets single dysfunctional mitochondria to the mitochondrial autophagy pathway [8]. And most importantly d) Mitochondrial fragmentation, mitochondrial outer membrane permeabilization, and cytochrome C release signals severely impaired host cells to undergo cell death [9]. This *mitochondrial ontology* is referred to as MQC and it is mediated by mitochondrial network dynamics. The mitochondrial network is regulated in highly sophisticated ways by various different posttranslational modifications, such as phosphorylation, ubiquitination, and proteolytic processing of its key-proteins, which also reflects the wide range of different intracellular and extracellular parameters integrated into the MQC [1,10,11]. Mitochondrial fission is facilitated by DRP1 [10], while MFN1 and MFN2 on the mitochondrial outer membrane, and OPA1 on the mitochondrial inner membrane are essential for mitochondrial fusion [1]. Mutations in most of the genes encoding MQC proteins have been associated with neurodegenerative diseases with tremendous effects on the whole organism (reviewed in [11] among others). Mutations in one of these genes, however, are associated with a neurodegenerative disease that originally was described to affect only the retinal ganglion cells, which connect the eye via the optic nerve to the brain. Though it is becoming more and more evident that other cell types are affected as well. In the following we would like to discuss the pathology of **d**ominant **o**ptic **a**trophy (DOA; OMIM: #165500) caused by heterozygous sequence variants in ***op****tic****a****trophy gene****1*** (OPA1; OMIM: *605290) in the light of the function of OPA1 in MQC. We think OPA1 is a good example to understand the molecular basis for MQC – the decision process between cell maintenance, mitochondrial autophagy, and cell death. We would like to point out, as mentioned above, that this represents only one part of mitochondrial network regulation among many and that we are just at the beginning of unraveling the complexity of this newly emerging field of MCQ.

### Dominant optic atrophy (DOA)

Neuropathies of the *nervus opticus* severely impair vision. One can distinguish between acquired optic neuropathies, which are mostly caused by intoxications (e.g. methanol, cyanide, lead, chloramphenicol, ethambutol) or nutritional deficiency symptoms (e.g. Vitamin B), and hereditary optic neuropathies, which can be further subdivided into syndromic forms with associated extra-ocular symptoms or non-syndromic forms limited to the ocular phenotype [12]. The two most common non-syndromic hereditary optic neuropathies are DOA with an estimated prevalence from 1:50 000 to 1:12 000 [13,14] and **L**eber’s **h**ereditary **o**ptic **n**europathy (LHON; OMIM: #535000). DOA is also referred to as **o**ptic **a**trophy, **K**jer type (OAK) or juvenile optic atrophy in older publications. DOA is associated with mutations in nuclear genes encoding mitochondrial proteins, primarily the *OPA1* gene [15,16], while LHON is associated with mutations in the remnant endosymbiotic genome, the mitochondrial DNA (mtDNA). Noteworthy, also acquired optic neuropathies involve mitochondrial impairments (cf. [17]). Glaucoma, the leading cause of worldwide blindness, is a non-syndromic optic neuropathy of the elderly and a complex disease associated with both environmental and genetic risk factors [18].

Patients with DOA suffer from slow progressive course of painless bilateral visual function loss with onset typically within the first two decades of life. The symptoms are mild to severe decrease in visual acuity, color vision deficiency, and visual field defects [19,20]. DOA is caused by loss of retinal ganglion cells only (RGCs) located in the inner retina and projecting their axons via the optic nerve to the brain. RGC loss and atrophy of the optic nerve are accompanied by thinning of the nerve fiber layer of the retina and the characteristic fundus with pallor of the optic disc [20,21], which is the structure where the RGC axons exit the eye. RGCs are the only affected cells among the 60 different neuronal cell types found in the retina and although photoreceptors are the cells with the highest oxygen consumption in the retina, light perception and signal processing in the retina is not impaired but signal transmission from the eye to the brain is distorted (see [22] and references therein). The clinical presentation of DOA is heterogeneous. The ocular phenotype is variable and not all family-members that carry pathogenic mutations in DOA associated genes present visual impairments [19-21,23-26]. The probability for mutation carriers to develop symptoms during lifetime has been estimated at 88% [26]. On the other hand, heterozygous *OPA1* mutations are associated with a broad range of extra-ocular symptoms, sometimes at the sub-clinical level. These symptoms include sensorineural deafness, ataxia, axonal sensory-motor polyneuropathy, chronic progressive external ophthalmoplegia, and mitochondrial myopathy [27-31]. Some studies therefore differentiate between non-syndromic and syndromic forms of DOA and suggest the later being associated with dominant-negative *OPA1* mutations [30,32]. However, one can observe the whole spectrum of disease manifestation from unaffected, to non-syndromic, to syndromic patients within one family segregating one single *OPA1* mutation [28,29]. This speaks more for a continuous clinical picture of DOA rather than a discrete one: different cell types are differently affected in different individuals with RGCs being mainly affected.

### Optic atrophy gene 1 (OPA1)

The human *OPA1* gene is composed of 30 coding exons (exon 1 to 28, exon 4b, exon 5b) distributed across more than 90 kb of genomic DNA on chromosome 3q28-q29. Alternative splicing of exons 4, 4b and 5b leads to eight isoforms with open reading frames for polypeptides of 924 to 1015 amino acids [33]. The OPA1 proteins are classified as large GTPases of the dynamin family, which are imported into mitochondria by their amino-terminal import sequence, and which are necessary for mitochondrial inner membrane fusion. With almost 300 sequence variants that cover the whole locus, *OPA1* is the most frequently mutated gene in DOA [34,35]. Mutations in *OPA1* account for at least 45% of all DOA cases and genomic rearrangements in the *OPA1* locus account for not less than an additional 10% of DOA cases [23,36].

*OPA1* is ubiquitously expressed and well conserved from yeast to man, which underpins its fundamental biological role. Genetic mouse- and fly-models that carry homozygous *OPA1* mutations show embryonic lethality [37,38], but OPA1-null mouse embryonic fibroblasts can be cultured [39], which suggests an essential function of mitochondrial inner membrane fusion during development [40]. Only very few patients carry confirmed compound heterozygous *OPA1* mutations, a 30-year-old woman (p.[E270K];[R290W]), who suffers from a severe ocular manifestation of DOA [41], a 60-year-old man and his 64-year-old sister (p.[S256R];[Q285R]), who both show ataxia, myopathy, peripheral neuropathy, and spasticity in addition to optic atrophy [42], an 8-year-old boy and his 3-year-old sister (p.[I382M];[V903Gfs*3]), who show severe optic atrophy already at this young age and severe neurological impairments with hypotonia and ataxia [43], and a 4-year-old boy (p.[S64fs];[V377I]), who also shows severe ocular phenotype already at this young age [44].

The OPA1 protein is associated with different functions, such as maintenance of the respiratory chain and membrane potential [45], cristae organization and control of apoptosis [46], as well as mitochondrial DNA maintenance [30,31,47]. And yet, all studies agree in the fact that OPA1 on the mitochondrial inner membrane, together with MFN1 and MFN2 on the mitochondrial outer membrane, is necessary for mitochondrial fusion and that this process is regulated by proteolytic cleavage of OPA1. Mitochondrial fusion in general requires both long OPA1 isoforms (OPA1L) and short OPA1 isoforms (OPA1S) [39], but the long OPA1L isoform alone is sufficient for stress-induced mitochondrial fusion [48]. Many different proteases directly or indirectly lead to OPA1 processing [39,49-54], among them are the **m**atrix– and the **i**ntermembrane space **A**TPases **a**ssociated with a number of cellular **a**ctivities (m-AAA and i-AAA protease, respectively), the **p**resenilin-**a**ssociated **r**homboid-**l**ike protease (PARL), the **h**igh **t**emperature **r**equirement **A2** protease (HTRA2) and **o**verlapping activity with **m**-**A**AA protease (OMA1), which all are associated with neurodegenerative diseases (reviewed in [11]). Of note, heterozygous mutations in ***s****pastic****p****araplegia****g****ene****7*** (*SPG7*; OMIM 602783), which codes for paraplegin, one of two monomers that assemble m-AAA proteases, have been identified in a four-generation family segregating non-syndromic DOA with no signs of spasticity, which is originally associated with mutations in *SPG7*[55].

Our current understanding of OPA1 processing is that OPA1 is translated in the cytosol and subsequently imported into mitochondria, where it is processed by the mitochondrial processing peptidase, which cleaves off the amino-terminal import sequence after amino acid position 87 (NP_056375) [51]. These long OPA1L isoforms are anchored to mitochondrial inner membrane and can be further processed at protease cleavage site S1 at amino acid position 195 (NP_056375) in exon 5, which results in short OPA1S isoforms devoid of the amino-terminal transmembrane domain [51]. Decrease of mitochondrial membrane potential ΔΨ_m_ results in OPA1 processing by OMA1, which cleaves all splice-forms of OPA1L at S1 in a ΔΨ_m_-dependent manner [53,54]. OPA1 splice-forms 4, 6, 7, and 8 include exon 5b, which contains an additional protease cleavage site S2 at around amino acid positions 217–223 (NP_570849) [39,51]. The mitochondrial i-AAA protease YME1L is necessary for proteolytic cleavage of OPA1L at S2 [39,50], and therefore generates OPA1S only from a subset of OPA1L isoforms, which allows adjustment of mitochondrial fusion (i.e. the ratio between OPA1L and OPA1S) by gene regulation as well as protein processing [11]. Knock-out of the mitochondrial inner membrane protein prohibitin by targeted deletion of the *Phb2* gene leads to mitochondrial fragmentation and abnormal cristae structure. This involves ΔΨ_m_–independent processing of OPA1L to OPA1S and can be rescued by expression of non-cleavable OPA1L [56]. OPA1 is involved in mitochondrial fusion and cristae remodeling [45]. Mitochondrial fusion and cristae remodeling are functionally distinct from each other and the later correlates with apoptotic cytochrome C release, which can be rescued by OPA1 overexpression [46]. Initially, PARL was suggested to be involved in OPA1L processing, cristae remodeling and subsequent cytochrome C release [52]. However, rhomboid proteases are not required for OPA1 processing [57] concealing the role of PARL in apoptosis (reviewed in [58]). Also the exact timing of cristae remodeling and cytochrome C release is still under debate (see [59] and references therein), as is the link between OPA1 and cristae junctions [46,60,61]. The only consent to date is that loss of OPA1 or inhibition of mitochondrial fusion by processing the entire pool of OPA1L and subsequent fragmentation of the mitochondrial network triggers cell death [39,45,50,51,53,54,56,57].

### Mitochondrial quality control (MQC)

**Figure 1:**
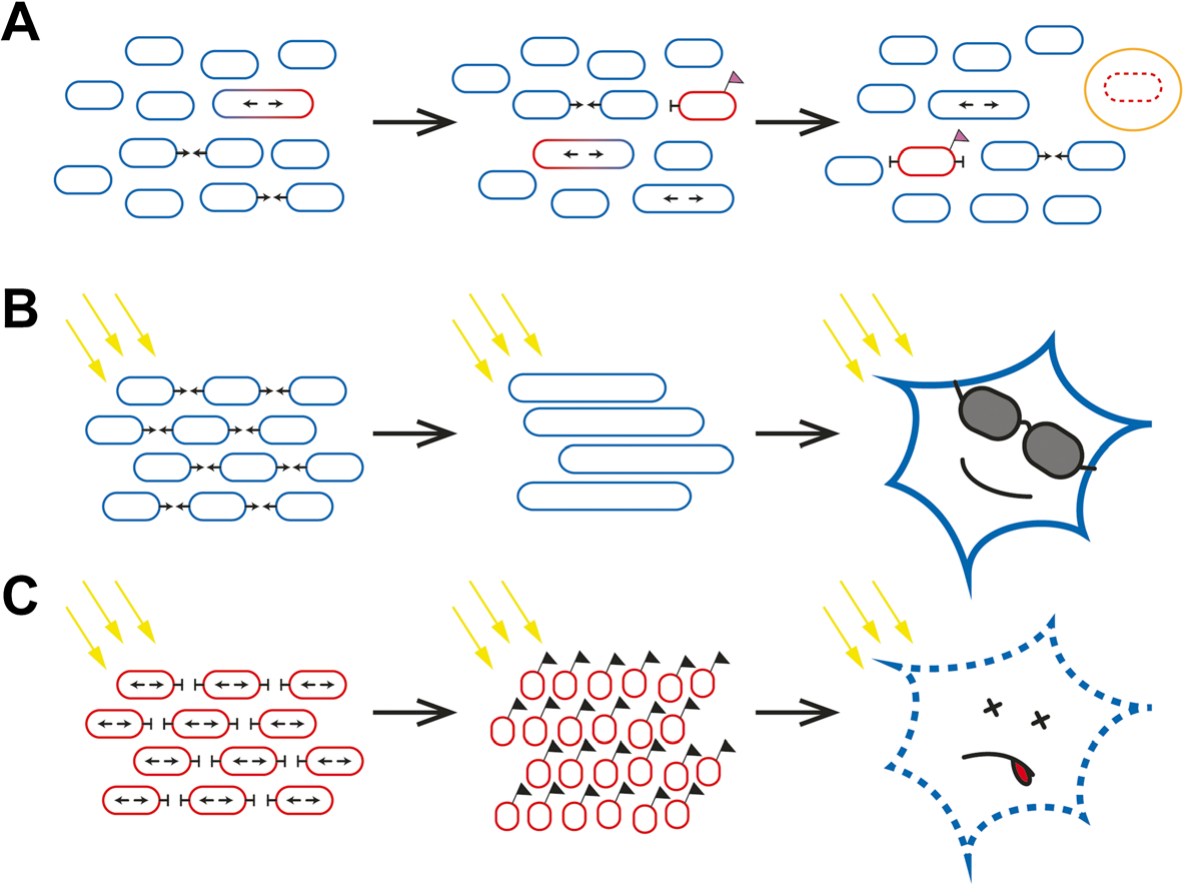
**Basic role of mitochondrial network dynamics in cell maintenance termed mitochondrial quality control.** Mitochondrial fission separates dysfunctional (red) from functional (blue) mitochondria. Refusion of dysfunctional mitochondria to the mitochondrial network is prevented and dysfunctional mitochondria are removed and recycled by mitochondrial autophagy **(A)**. If a cell is stressed (yellow arrows in **B** and **C**), mitochondria fuse in order to protect themselves and to warrant that they are still functional **(B)**. If the stress prevails, mitochondrial fusion is prevented, mitochondria fragment, and mitochondria signal their stressed host cell to undergo cell death thereby removing the whole cell from the organism **(C)**.

Mitochondria are vulnerable to damage of their proteins, lipids, and mtDNA caused by various stress factors and mitochondrial fusion allows for exchange of mtDNA [7] and mitochondrial content between organelles in order to attenuate or complement this damage [9]. In addition, mitochondrial fission and selective fusion is able to separate functional from dysfunctional mitochondria; single dysfunctional mitochondria are sorted out and degraded by mitochondrial autophagy (see Figure 1A). A study on pancreatic β cells has shown that mitochondrial fission generates uneven daughter units with respect to their membrane potential [8]. Mitochondria with reduced membrane potential are prevented from re-fusing to the mitochondrial network by different mechanisms [9]. One of these mechanisms is inhibition of mitochondrial fusion by ΔΨ_m_-dependent proteolytic cleavage of OPA1L isoforms [8]. Single mitochondria that don’t fuse anymore are subsequently targeted to mitochondrial autophagy and in line with this *Opa1* mutant mice show increased autophagy in the optic nerve [62]. Conversely, it has been shown that during starvation, mitochondria elongate and are spared from autophagy [5]. Targeting of mitochondria with reduced membrane potential to autophagosomes is accomplished by the PINK1/Parkin pathway, which is associated with familial Parkinsonism [63]. Together, these findings document that mitochondrial fusion, apart from its role in exchanging mtDNA and mitochondrial content, plays a fundamental role in maintenance of mitochondria.

Mitochondrial network dynamics are regulated in highly sophisticated ways by various posttranslational modifications and proteolytic processing of the key-proteins DRP1, MFN1 and MFN2, and OPA1 [10,11]. The carefully regulated balance of mitochondrial fusion and fission integrates not only the mitochondrial membrane potential, but also other mitochondrial, as well as intracellular and extracellular parameters, such as redox state, nutrition state, and toxicity load [48]. Mitochondria can cope with these stress factors to certain extends by fusing mitochondria and forming very long connected tubular mitochondria (Figure 1B). However, if the stressors prevail and mitochondrial damage is too high, mitochondria signal the eukaryotic host cell to undergo cell death by mitochondrial fragmentation, mitochondrial outer membrane permeabilization, and cytochrome C release into the cytosol (Figure 1C). OPA1 is involved in mitochondrial fusion and cytochrome C release [45], which are functionally distinct [46], and therefore the capability of mitochondria to fuse their inner membrane and not fusion itself – in other words the fusion competent long isoform OPA1L – is crucial to counteract this cell death signal cascade [56]. In line with this is the anti-apoptotic function of **h**ypoxia-**i**nduced **g**ene **d**omain protein-**1a** (Higd-1a), which was shown to bind OPA1 and in doing so to prevent proteolytic cleavage of OPA1L to OPA1S, which in turn counteracted cytochrome C release [64,65]. Of note, stress-induced mitochondrial hyperfusion also relies only on the long OPA1L isoforms [48]. But still the molecular basis for the anti-apoptotic function of OPA1L is not fully resolved yet.

**Figure 2:**
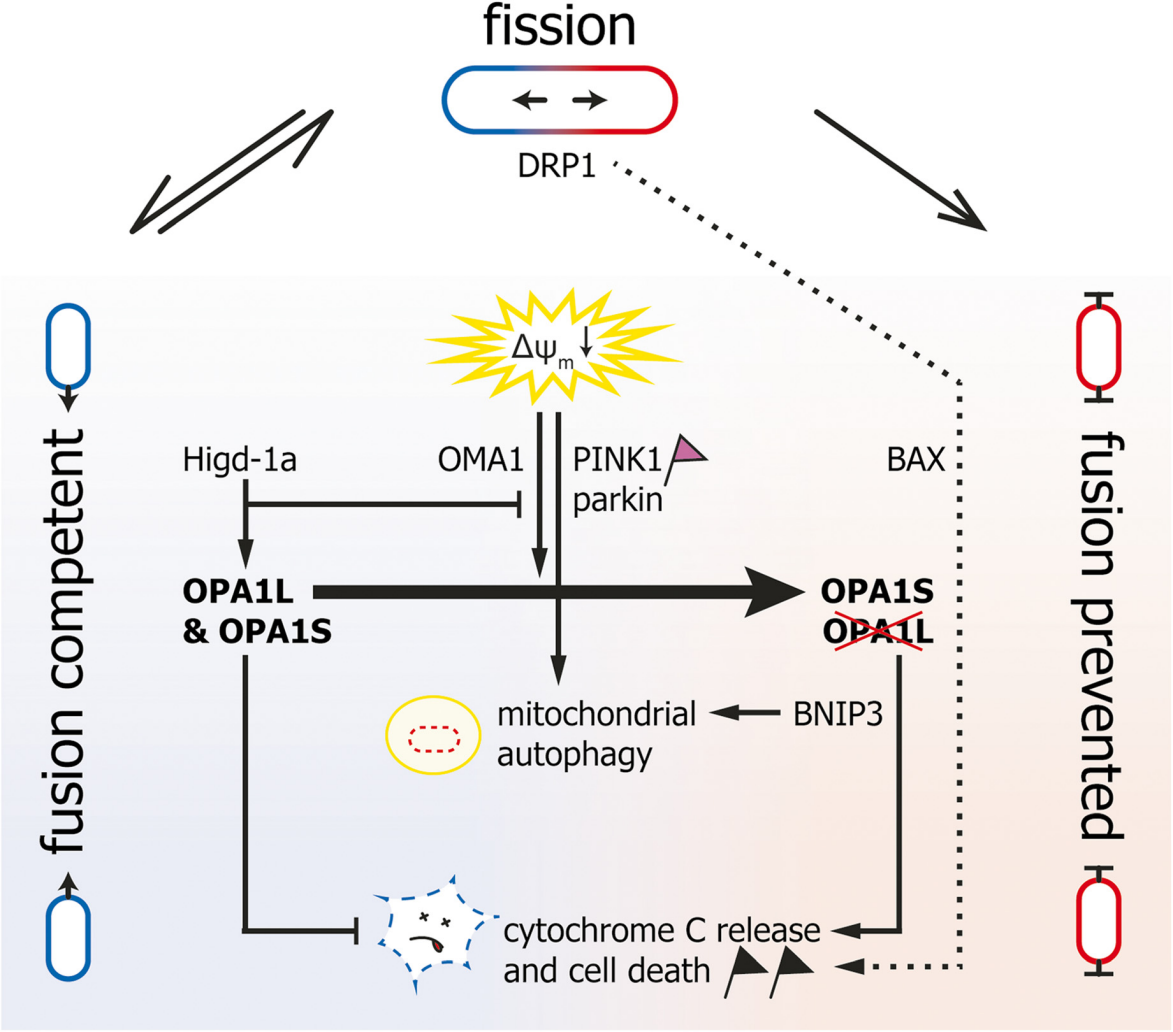
**Principle of mitochondrial quality control with focus on OPA1 function and its contribution to mitochondrial network dynamics.** DRP1 recruitment to mitochondria is necessary and sufficient for mitochondrial fission. In healthy mitochondria there is a continuous equilibrium between mitochondrial fission (top) and fusion (left side). Dysfunctional mitochondria can be repaired and rescued by re-fusion to the mitochondrial network (not depicted for the sake of clarity). OPA1L and OPA1S (together with MFN1/2 on the mitochondrial outer membrane) are necessary and sufficient for maintaining fusion competent mitochondria. Fusion is prevented in dysfunctional mitochondria by proteolytic cleavage of OPA1L to OPA1S (right side). The best-characterized example of dysfunctional mitochondria so far is dissipation of the mitochondrial membrane potential (ΔΨ_m_), which leads to proteolytic cleavage of OPA1L by OMA1. OPA1 cleavage, however, occurs under various conditions enabling the integration of various parameters into mitochondrial network dynamics. Higd-1a, for example, regulates mitochondrial network dynamics by binding to OPA1L and thereby inhibiting its proteolytic cleavage. Dissipation of the mitochondrial membrane potential activates in the long run also the PINK1/parkin pathway (pink flag), which targets dysfunctional mitochondria for mitochondrial autophagy. OPA1L counteracts cytochrome C release and cell death (black flag) and therefore acts anti-apoptotic. OPA1S (presumably together with BNIP3) is necessary to promote cytochrome C release and cell death. This outer membrane permeabilization might be independent or happen in cooperation with BAX and DRP1 dependent outer membrane permeabilization (dotted line). BNIP3 is also necessary for mitochondrial autophagy and therefore could play an important role in the decision whether to remove single dysfunctional mitochondria or to recycle the whole cell by undergoing cell death.

Mitochondrial outer membrane permeabilization in order to release cytochrome C with subsequent cell death is facilitated by various mechanisms [9,66-69]. Of note in this context is that cardiomyocytes from *Opa1* mutant mice display delayed permeability transition pore opening under calcium stimulation and therefore a higher calcium retention capacity [70]. This indicates that OPA1 – whether the fusion competent long OPA1L isoforms or the fusion incompetent short OPA1S isoforms is still open – might be involved directly in mitochondrial outer membrane permeabilization. Therefore OPA1 could have also a pro-cell death function besides its anti-apoptotic function described above. BNIP3, a mitochondrial pro-apoptotic BH3-only protein of the BCL2 family, is a direct interaction partner of OPA1 [71], which has been associated with an alternative mechanism of mitochondrial outer membrane permeabilization [72]. This substantiates the pro-cell death function of OPA1(S). Figure 2 gives a simplified summary of the discussed MQC pathways and key-proteins with focus on the role of OPA1 processing.

## Discussion

It seems rather complicated to understand mitochondrial network morphology and its significance to disease pathology. However, this relation might become clearer if one recalls that *the eukaryotic cell can ultimately be understood only on the basis of its history*[73]. Mitochondria are descendants of a primary endosymbiosis almost 2 billion years ago. And although mitochondria have transferred almost their entire genome to the nucleus of their host, mitochondria have still preserved the power to break up the alliance with their host by signaling cell death, something we refer to as apoptosis. Mitochondria achieve this by releasing cytochrome C into the cytoplasm of their host and this strategy confers an evolutionary advantage to higher eukaryotes although, by doing so, mitochondria extinguish themselves together with their host. This becomes more plausible if one considers that mitochondria take many different intracellular and extracellular parameters into account before they decide to quit the liaison with their host. As long as mitochondria are happy, they continue to function normally supplying their host with various metabolites and energy. Once the mitochondrial environment – in other words the host cell – is inappropriate, mitochondria release cytochrome C and signal cell death. In addition, single dysfunctional or damaged mitochondria can be removed from the mitochondrial pool by mitochondrial autophagy without any harm for the host cell. This whole process is called MQC (though *mitochondrial ontology* would be more to the point).

**Figure 3:**
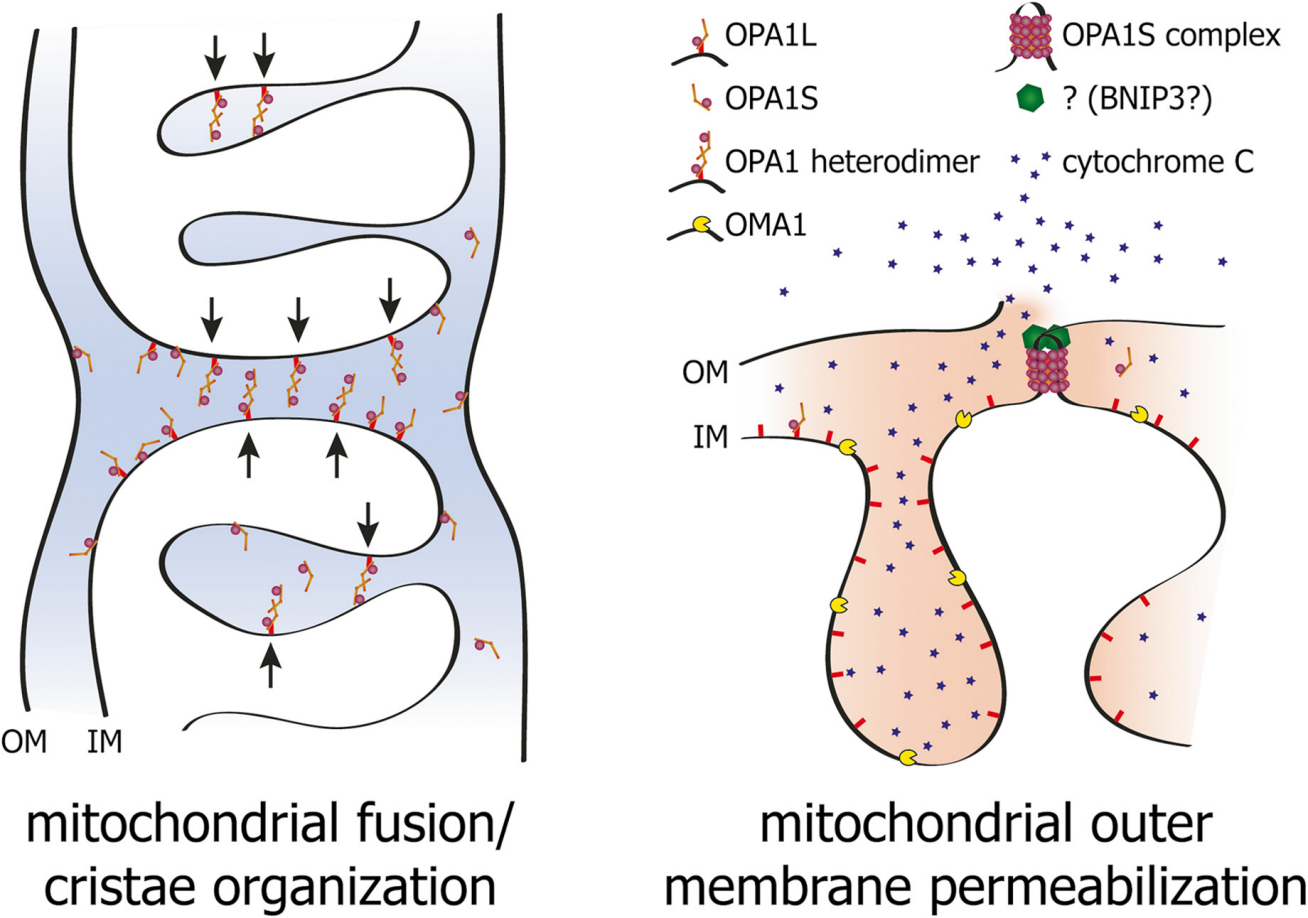
**Molecular model of the dual function of OPA1 in mitochondrial inner membrane (IM) fusion and cytochrome C release and cell death.** Membrane bound OPA1L and soluble OPA1S form a complex that is able to attract two mitochondrial membranes to enable inner membrane fusion (left) and cristae organization. Proteolytic cleavage of OPA1L to the soluble OPA1S allows the formation of OPA1S complex, which together with BNIP3 promotes mitochondrial outer membrane (OM) permeabilization, cytochrome C release, and cell death (right) by bringing the mitochondrial inner membrane in proximity to the mitochondrial outer membrane, thereby facilitating membrane hemi-fusion states that are energetic favorable for membrane permeabilization.

One model to explain the apparently contradictory finding of an anti-apoptotic as well as pro-cell death function of OPA1 is that OPA1S supports outer membrane permeabilization by generating membrane hemi-fusion intermediates between the mitochondrial inner– and outer membrane (Figure 3). This confers a pro-cell death function to OPA1 because hemi-fusion intermediates are energetically favorable for membrane permeabilization. Similar was suggested previously for hemi-fission states of mitochondrial outer membranes [74], or hemi-fusion intermediates of the mitochondrial outer membrane and the endoplasmatic reticulum [67] and it might depend on the cell type and the nature of stress factors which mechanism of mitochondrial outer membrane permeabilization dominates. The inner membrane bound OPA1L on the other hand binds the soluble OPA1S in order to enable mitochondrial inner membrane fusion, thereby preventing inner membrane and outer membrane hemi-fusion intermediates, which explains the anti-apoptotic function of OPA1L. The pro-cell death and anti-apoptotic contributions of OPA1 might differ between cell types and depend on the nature of the stress factor. One could speculate that for neuronal cells DRP1 and BAX are more relevant for outer membrane permeabilization, while in reperfusion of cardiomyocytes outer membrane permeabilization depends more on OPA1 and BNIP3. This model of various alternative mechanisms for outer membrane permeabilization can explain the broad range of cell death between the two extremes necrotic- and apoptotic cell death.

Mitochondrial fusion and fission integrates not only the mitochondrial membrane potential ΔΨ_m_; OPA1L is processed under various stress conditions, such as redox state, nutrition state, and toxicity load. This implies that mitochondrial targeting to autophagy is not only accomplished by the ΔΨ_m_ dependent PINK1/parkin pathway, but also by other pathways. One such pathway could involve BNIP3, because BNIP3 interacts directly with the autophagy machinery [75] and is necessary for mitochondrial autophagy induced by hypoxia [76]. Interestingly, BNIP3 is also highly expressed in neuronal models of excitotoxicity [77], which is a pathological incident that leads to neuronal cell death by excessive neurotransmitter stimulation, for example by stimulation of ionotropic glutamate receptors, such as NMDA receptors. *Opa1* mutant mice show extensive mitochondrial fission in RGC axons of the optic nerve head and significantly increased NMDA receptor expression in the retina [78]. On the other hand it was shown that excitotoxicity results in mitochondrial hyper-fragmentation by the activation of the DRP1 fission pathway [79]. This shows that excitotoxicity can modify the mitochondrial network and vice versa the mitochondrial network alters components involved in excitotoxicity and this vicious cycle likely contributes to disease pathology in DOA.

*Opa1* mutant mice resemble the human disease phenotype [22,38]. They also show reduced levels of the mitochondrial superoxide dismutase SOD2, which disposes toxic superoxide into hydrogen peroxide and oxygen [78]. Increased ROS has been found in *OPA1* mutant worms and flies [80-82], and OPA1 seems to alter mitochondrial respiration in patients with DOA [83,84]. The integration of mitochondrial respiration and reactive oxygen species (ROS) into the regulation of mitochondrial fission and fusion, however, is still not fully resolved. Noteworthy in this context is that OPA1L processing is inhibited by Higd-1a and silencing of Higd-1a results in growth retardation, cristae disorganization and loss of mtDNA [65]. Loss of mtDNA was also described in patients with non-syndromic DOA [85] and in patients with syndromic DOA, where loss of mtDNA caused cytochrome C oxidase deficiency [86]. OPA1 exon 4b is thought to alter mtDNA replication and distribution [47]. Also mtDNA deletions have been associated with *OPA1* mutations in patients with non-syndromic DOA [42] and syndromic DOA [30,31]. Conditional MFN1 and MFN2 double knock-out mice also accumulate mtDNA deletions and show severe mtDNA depletion [87]. However, other studies challenge a direct link of OPA1 and mtDNA deletions [88,89]. Particularly patients with severe course of disease revealed no changes in mtDNA, for example one patient carrying an *OPA1* mutation and diagnosed with Behr syndrome [90], as well as the two children that carry compound heterozygous OPA1 mutations [43]. Data on the three *Opa1* mouse models are consistent with these findings because optic nerve dysfunction and extra-ocular impairments in these models are not linked with mtDNA deletions or mtDNA depletion either [32,91,92]. To conclude, loss of mtDNA or mtDNA deletions can account for some but not all of the phenotypes associated with *OPA1* mutations. Although tissue specific compensation of mtDNA alterations might be an explanation [32], mtDNA deletions and mtDNA depletion could as well be the consequence of impaired MQC rather than the primary cause of the disease.

Recently OPA1 was found associated to lipid droplets of murine adipocytes and this study demonstrated that silencing of OPA1 affects the adrenergic regulation of the lipolysis [93]. In this context it is noteworthy that 20-month old *Opa1* mutant mice showed no signs of obesity under regular animal housing conditions, while all animals in the control group were morbidly obese at this age [91]. Cardiac mitochondria of *Opa1* mutant mice are also less able to oxidize lipids compared to mitochondria of control mice [70]. Mice lacking the protease *Oma1*, which has an inhibitory effect on OPA1 by processing OPA1L at S1, are obese and show decreased fatty-acid β-oxidation compared to controls [94]. These findings imply that OPA1 is also directly involved in the integration of the cellular metabolic state into the mitochondrial network and it is well established that mitochondrial network morphology changes to adapt oxidative phosphorylation to the metabolic state of the cell [6]. In accordance to this are the alterations of oxidative phosphorylation observed in patients with DOA and OPA1-silenced cells [60,86]. Still, the knowledge on the molecular basis for the integration of mitochondrial or cellular parameters other than ΔΨ_m_ in MQC is still vague and this topic deserves more exploration.

## Conclusions

Mitochondrial fission and fusion occupies a central position in MQC and it processes different intracellular and extracellular parameters in order to accommodate cell homeostasis and cell fate by the following measures: a) Mitochondrial network morphology changes to either a more filamentous or more fragmented state to adapt mitochondrial function to actual energetic and metabolic demands of the host cell. b) Fusion of dysfunctional mitochondria to functional mitochondria can attenuate damage to mitochondrial proteins, lipids, and mtDNA. c) Inhibition of mitochondrial fusion targets single dysfunctional mitochondria to the mitochondrial autophagy pathway. d) Mitochondrial fragmentation, mitochondrial outer membrane permeabilization, and cytochrome C release signals severely impaired host cells to undergo cell death. According to this, mitochondrial fusion maintains mitochondria by attenuating mitochondrial damage as well as by protecting from mitochondrial autophagy. Moreover mitochondrial fusion antagonizes cell death signaling. That is why reducing the ability of mitochondria to fuse – as implied by mutations in *OPA1* or deletion of one *OPA1* allele – compromises MQC in a way that cells are more prone to intracellular and extracellular stress factors. Interestingly this means that dominant-negative *OPA1* mutations can lead to the removal of the protein coded by this dominant-negative *OPA1* allele by boosting mitochondrial autophagy. This can phenocopy haploinsufficiency, since mutant protein would not be detectable unless one interferes pharmacologically with MQC. Additionally, dominant-negative *OPA1* mutations can involve also higher mitochondrial turnover rates as amplified mitochondrial biogenesis may balance higher mitochondrial autophagy rates.

Impairments in MQC affect different cell types in different individuals in a different way depending on the individual’s unique profile of intracellular and extracellular stress factors. In other words, the heterogeneous clinical presentation of patients with DOA is caused by the individual’s unique profile of genetic and environmental risk factors. Then one can ask which risk factors do harm RGCs more than other cell types? These risk factors are possibly the same that lead to RGC death in glaucoma and genetic and environmental risk factors that trigger glaucoma are reviewed in numerous publications and are beyond the scope of this article. And yet, also *OPA1* polymorphisms are discussed to be associated with certain forms of glaucoma [95]. In this context, it is of particular interest that glaucomatous insults trigger OPA1 cleavage, mitochondrial fission, and mitochondrial autophagy in RGC axons of the optic nerve head in a mouse model of glaucoma [96].

To sum up, *OPA1* mutations impair MQC, which is more *mitochondrial ontology* than quality control, thereby rendering cells more susceptible to stress factors. In particular RGCs are under risk but all other cell types can be affected, too. Looking at glaucoma, there seems to be a unique risk profile for RGCs, which is also applicable to DOA. This brings together DOA and glaucoma.

## Competing interests

The authors have no competing financial interests.

## Authors’ contributions

MVA and NF jointly developed ideas and wrote the manuscript. MVA prepared the figures. Both authors read and approved the final manuscript.

## References

[B1] Cho DH Nakamura T Lipton SA Mitochondrial dynamics in cell death and neurodegeneration Cell Mol Life Sci 2010 67 20 3435 3447 20577776 10.1007/s00018-010-0435-2 PMC11115814

[B2] Piquereau J Caffin F Novotova M Lemaire C Veksler V Garnier A Ventura-Clapier R Joubert F Mitochondrial dynamics in the adult cardiomyocytes: which roles for a highly specialized cell? Front Physiol 2013 4 102 23675354 10.3389/fphys.2013.00102 PMC3650619

[B3] Grandemange S Herzig S Martinou JC Mitochondrial dynamics and cancer Semin Cancer Biol 2009 19 1 50 56 19138741 10.1016/j.semcancer.2008.12.001

[B4] Wang W Lu J Zhu F Wei J Jia C Zhang Y Zhou L Xie H Zheng S Pro-apoptotic and anti-proliferative effects of mitofusin-2 via Bax signaling in hepatocellular carcinoma cells Med Oncol 2012 29 1 70 76 21190094 10.1007/s12032-010-9779-6

[B5] Gomes LC Di Benedetto G Scorrano L During autophagy mitochondria elongate, are spared from degradation and sustain cell viability Nat Cell Biol 2011 13 5 589 598 21478857 10.1038/ncb2220 PMC3088644

[B6] Hackenbrock CR Chemical and physical fixation of isolated mitochondria in low-energy and high-energy states Proc Natl Acad Sci U S A 1968 61 2 598 605 4176482 10.1073/pnas.61.2.598 PMC225202

[B7] Yoneda M Miyatake T Attardi G Complementation of mutant and wild-type human mitochondrial DNAs coexisting since the mutation event and lack of complementation of DNAs introduced separately into a cell within distinct organelles Mol Cell Biol 1994 14 4 2699 2712 8139569 10.1128/mcb.14.4.2699-2712.1994 PMC358636

[B8] Twig G Elorza A Molina AJ Mohamed H Wikstrom JD Walzer G Stiles L Haigh SE Katz S Las G Fission and selective fusion govern mitochondrial segregation and elimination by autophagy EMBO J 2008 27 2 433 446 18200046 10.1038/sj.emboj.7601963 PMC2234339

[B9] Youle RJ van der Bliek AM Mitochondrial fission, fusion, and stress Science 2012 337 6098 1062 1065 22936770 10.1126/science.1219855 PMC4762028

[B10] Chang CR Blackstone C Dynamic regulation of mitochondrial fission through modification of the dynamin-related protein Drp1 Ann N Y Acad Sci 2010 1201 34 39 20649536 10.1111/j.1749-6632.2010.05629.x PMC5585781

[B11] Rugarli EI Langer T Mitochondrial quality control: a matter of life and death for neurons EMBO J 2012 31 6 1336 1349 22354038 10.1038/emboj.2012.38 PMC3321185

[B12] Carelli V Ross-Cisneros FN Sadun AA Mitochondrial dysfunction as a cause of optic neuropathies Prog Retin Eye Res 2004 23 1 53 89 14766317 10.1016/j.preteyeres.2003.10.003

[B13] Kivlin JD Lovrien EW Bishop DT Maumenee IH Linkage analysis in dominant optic atrophy Am J Hum Genet 1983 35 6 1190 1195 6580816 PMC1685968

[B14] Kjer B Eiberg H Kjer P Rosenberg T Dominant optic atrophy mapped to chromosome 3q region. II. Clinical and epidemiological aspects Acta Ophthalmol Scand 1996 74 1 3 7 8689476 10.1111/j.1600-0420.1996.tb00672.x

[B15] Alexander C Votruba M Pesch UE Thiselton DL Mayer S Moore A Rodriguez M Kellner U Leo-Kottler B Auburger G OPA1, encoding a dynamin-related GTPase, is mutated in autosomal dominant optic atrophy linked to chromosome 3q28 Nat Genet 2000 26 2 211 215 11017080 10.1038/79944

[B16] Delettre C Lenaers G Griffoin JM Gigarel N Lorenzo C Belenguer P Pelloquin L Grosgeorge J Turc-Carel C Perret E Nuclear gene OPA1, encoding a mitochondrial dynamin-related protein, is mutated in dominant optic atrophy Nat Genet 2000 26 2 207 210 11017079 10.1038/79936

[B17] Sadun AA Mitochondrial optic neuropathies J Neurol Neurosurg Psychiatry 2002 72 4 423 425 11909893 10.1136/jnnp.72.4.423 PMC1737836

[B18] Quigley HA Glaucoma Lancet 2011 377 9774 1367 1377 21453963 10.1016/S0140-6736(10)61423-7

[B19] Hoyt CS Autosomal dominant optic atrophy. A spectrum of disability Ophthalmology 1980 87 3 245 251 7422264 10.1016/s0161-6420(80)35247-0

[B20] Kline LB Glaser JS Dominant optic atrophy. The clinical profile Arch Ophthalmol 1979 97 9 1680 1686 314284 10.1001/archopht.1979.01020020248013

[B21] Cohn AC Toomes C Potter C Towns KV Hewitt AW Inglehearn CF Craig JE Mackey DA Autosomal dominant optic atrophy: penetrance and expressivity in patients with OPA1 mutations Am J Ophthalmol 2007 143 4 656 662 17306754 10.1016/j.ajo.2006.12.038

[B22] Heiduschka P Schnichels S Fuhrmann N Hofmeister S Schraermeyer U Wissinger B Alavi MV Electrophysiological and histologic assessment of retinal ganglion cell fate in a mouse model for OPA1-associated autosomal dominant optic atrophy Invest Ophthalmol Vis Sci 2010 51 3 1424 1431 19834041 10.1167/iovs.09-3606

[B23] Fuhrmann N Alavi MV Bitoun P Woernle S Auburger G Leo-Kottler B Yu-Wai-Man P Chinnery P Wissinger B Genomic rearrangements in OPA1 are frequent in patients with autosomal dominant optic atrophy J Med Genet 2009 46 2 136 144 19181907 10.1136/jmg.2008.062570

[B24] Marchbank NJ Craig JE Leek JP Toohey M Churchill AJ Markham AF Mackey DA Toomes C Inglehearn CF Deletion of the OPA1 gene in a dominant optic atrophy family: evidence that haploinsufficiency is the cause of disease J Med Genet 2002 39 8 e47 12161614 10.1136/jmg.39.8.e47 PMC1735190

[B25] Puomila A Huoponen K Mantyjarvi M Hamalainen P Paananen R Sankila EM Savontaus ML Somer M Nikoskelainen E Dominant optic atrophy: correlation between clinical and molecular genetic studies Acta Ophthalmol Scand 2005 83 3 337 346 15948788 10.1111/j.1600-0420.2005.00448.x

[B26] Fuhrmann N Schimpf S Kamenisch Y Leo-Kottler B Alexander C Auburger G Zrenner E Wissinger B Alavi MV Solving a 50 year mystery of a missing OPA1 mutation: more insights from the first family diagnosed with autosomal dominant optic atrophy Mol Neurodegener 2010 5 1 25 20546606 10.1186/1750-1326-5-25 PMC2893178

[B27] Amati-Bonneau P Odent S Derrien C Pasquier L Malthiery Y Reynier P Bonneau D The association of autosomal dominant optic atrophy and moderate deafness may be due to the R445H mutation in the OPA1 gene Am J Ophthalmol 2003 136 6 1170 1171 14644237 10.1016/s0002-9394(03)00665-2

[B28] Li C Kosmorsky G Zhang K Katz BJ Ge J Traboulsi EI Optic atrophy and sensorineural hearing loss in a family caused by an R445H OPA1 mutation Am J Med Genet A 2005 138 3 208 211 16158427 10.1002/ajmg.a.30794

[B29] Payne M Yang Z Katz BJ Warner JE Weight CJ Zhao Y Pearson ED Treft RL Hillman T Kennedy RJ Dominant optic atrophy, sensorineural hearing loss, ptosis, and ophthalmoplegia: a syndrome caused by a missense mutation in OPA1 Am J Ophthalmol 2004 138 5 749 755 15531309 10.1016/j.ajo.2004.06.011

[B30] Amati-Bonneau P Valentino ML Reynier P Gallardo ME Bornstein B Boissiere A Campos Y Rivera H de la Aleja JG Carroccia R OPA1 mutations induce mitochondrial DNA instability and optic atrophy 'plus' phenotypes Brain 2008 131 Pt 2 338 351 18158317 10.1093/brain/awm298

[B31] Hudson G Amati-Bonneau P Blakely EL Stewart JD He L Schaefer AM Griffiths PG Ahlqvist K Suomalainen A Reynier P Mutation of OPA1 causes dominant optic atrophy with external ophthalmoplegia, ataxia, deafness and multiple mitochondrial DNA deletions: a novel disorder of mtDNA maintenance Brain 2008 131 Pt 2 329 337 18065439 10.1093/brain/awm272

[B32] Sarzi E Angebault C Seveno M Gueguen N Chaix B Bielicki G Boddaert N Mausset-Bonnefont AL Cazevieille C Rigau V The human OPA1delTTAG mutation induces premature age-related systemic neurodegeneration in mouse Brain 2012 135 Pt 12 3599 3613 23250881 10.1093/brain/aws303

[B33] Delettre C Griffoin JM Kaplan J Dollfus H Lorenz B Faivre L Lenaers G Belenguer P Hamel CP Mutation spectrum and splicing variants in the OPA1 gene Hum Genet 2001 109 6 584 591 11810270 10.1007/s00439-001-0633-y

[B34] Ferre M Amati-Bonneau P Tourmen Y Malthiery Y Reynier P eOPA1: an online database for OPA1 mutations Hum Mutat 2005 25 5 423 428 15832306 10.1002/humu.20161

[B35] MITOchondrial DYNamics variation pages MITOchondrial DYNamics variation pages http://mitodyn.org/home.php

[B36] Almind GJ Gronskov K Milea D Larsen M Brondum-Nielsen K Ek J Genomic deletions in OPA1 in Danish patients with autosomal dominant optic atrophy BMC Med Genet 2011 12 49 21457585 10.1186/1471-2350-12-49 PMC3079616

[B37] McQuibban GA Lee JR Zheng L Juusola M Freeman M Normal mitochondrial dynamics requires rhomboid-7 and affects Drosophila lifespan and neuronal function Curr Biol 2006 16 10 982 989 16713954 10.1016/j.cub.2006.03.062

[B38] Alavi MV Bette S Schimpf S Schuettauf F Schraermeyer U Wehrl HF Ruttiger L Beck SC Tonagel F Pichler BJ A splice site mutation in the murine Opa1 gene features pathology of autosomal dominant optic atrophy Brain 2007 130 Pt 4 1029 1042 17314202 10.1093/brain/awm005

[B39] Song Z Chen H Fiket M Alexander C Chan DC OPA1 processing controls mitochondrial fusion and is regulated by mRNA splicing, membrane potential, and Yme1L J Cell Biol 2007 178 5 749 755 17709429 10.1083/jcb.200704110 PMC2064540

[B40] Rahn JJ Stackley KD Chan SS Opa1 is required for proper mitochondrial metabolism in early development PLoS One 2013 8 3 e59218 23516612 10.1371/journal.pone.0059218 PMC3597633

[B41] Pesch UE Leo-Kottler B Mayer S Jurklies B Kellner U Apfelstedt-Sylla E Zrenner E Alexander C Wissinger B OPA1 mutations in patients with autosomal dominant optic atrophy and evidence for semi-dominant inheritance Hum Mol Genet 2001 10 13 1359 1368 11440988 10.1093/hmg/10.13.1359

[B42] Yu-Wai-Man P Griffiths PG Gorman GS Lourenco CM Wright AF Auer-Grumbach M Toscano A Musumeci O Valentino ML Caporali L Multi-system neurological disease is common in patients with OPA1 mutations Brain 2010 133 Pt 3 771 786 20157015 10.1093/brain/awq007 PMC2842512

[B43] Schaaf CP Blazo M Lewis RA Tonini RE Takei H Wang J Wong LJ Scaglia F Early-onset severe neuromuscular phenotype associated with compound heterozygosity for OPA1 mutations Mol Genet Metab 2011 103 4 383 387 21636302 10.1016/j.ymgme.2011.04.018

[B44] Chen Y Jia X Wang P Xiao X Li S Guo X Zhang Q Mutation survey of the optic atrophy 1 gene in 193 Chinese families with suspected hereditary optic neuropathy Mol Vis 2013 19 292 302 23401657 PMC3566897

[B45] Olichon A Baricault L Gas N Guillou E Valette A Belenguer P Lenaers G Loss of OPA1 perturbates the mitochondrial inner membrane structure and integrity, leading to cytochrome c release and apoptosis J Biol Chem 2003 278 10 7743 7746 12509422 10.1074/jbc.C200677200

[B46] Frezza C Cipolat S Martins de Brito O Micaroni M Beznoussenko GV Rudka T Bartoli D Polishuck RS Danial NN De Strooper B Scorrano L OPA1 controls apoptotic cristae remodeling independently from mitochondrial fusion Cell 2006 126 1 177 189 16839885 10.1016/j.cell.2006.06.025

[B47] Elachouri G Vidoni S Zanna C Pattyn A Boukhaddaoui H Gaget K Yu-Wai-Man P Gasparre G Sarzi E Delettre C OPA1 links human mitochondrial genome maintenance to mtDNA replication and distribution Genome Res 2011 21 1 12 20 20974897 10.1101/gr.108696.110 PMC3012919

[B48] Tondera D Grandemange S Jourdain A Karbowski M Mattenberger Y Herzig S Da Cruz S Clerc P Raschke I Merkwirth C SLP-2 is required for stress-induced mitochondrial hyperfusion EMBO J 2009 28 11 1589 1600 19360003 10.1038/emboj.2009.89 PMC2693158

[B49] Duvezin-Caubet S Jagasia R Wagener J Hofmann S Trifunovic A Hansson A Chomyn A Bauer MF Attardi G Larsson NG Proteolytic processing of OPA1 links mitochondrial dysfunction to alterations in mitochondrial morphology J Biol Chem 2006 281 49 37972 37979 17003040 10.1074/jbc.M606059200

[B50] Griparic L Kanazawa T van der Bliek AM Regulation of the mitochondrial dynamin-like protein Opa1 by proteolytic cleavage J Cell Biol 2007 178 5 757 764 17709430 10.1083/jcb.200704112 PMC2064541

[B51] Ishihara N Fujita Y Oka T Mihara K Regulation of mitochondrial morphology through proteolytic cleavage of OPA1 Embo J 2006 25 13 2966 2977 16778770 10.1038/sj.emboj.7601184 PMC1500981

[B52] Cipolat S Rudka T Hartmann D Costa V Serneels L Craessaerts K Metzger K Frezza C Annaert W D'Adamio L Mitochondrial rhomboid PARL regulates cytochrome c release during apoptosis via OPA1-dependent cristae remodeling Cell 2006 126 1 163 175 16839884 10.1016/j.cell.2006.06.021

[B53] Ehses S Raschke I Mancuso G Bernacchia A Geimer S Tondera D Martinou JC Westermann B Rugarli EI Langer T Regulation of OPA1 processing and mitochondrial fusion by m-AAA protease isoenzymes and OMA1 J Cell Biol 2009 187 7 1023 1036 20038678 10.1083/jcb.200906084 PMC2806285

[B54] Head B Griparic L Amiri M Gandre-Babbe S van der Bliek AM Inducible proteolytic inactivation of OPA1 mediated by the OMA1 protease in mammalian cells J Cell Biol 2009 187 7 959 966 20038677 10.1083/jcb.200906083 PMC2806274

[B55] Klebe S Depienne C Gerber S Challe G Anheim M Charles P Fedirko E Lejeune E Cottineau J Brusco A Spastic paraplegia gene 7 in patients with spasticity and/or optic neuropathy Brain 2012 135 Pt 10 2980 2993 23065789 10.1093/brain/aws240 PMC3470714

[B56] Merkwirth C Dargazanli S Tatsuta T Geimer S Lower B Wunderlich FT von Kleist-Retzow JC Waisman A Westermann B Langer T Prohibitins control cell proliferation and apoptosis by regulating OPA1-dependent cristae morphogenesis in mitochondria Genes Dev 2008 22 4 476 488 18281461 10.1101/gad.460708 PMC2238669

[B57] Duvezin-Caubet S Koppen M Wagener J Zick M Israel L Bernacchia A Jagasia R Rugarli EI Imhof A Neupert W OPA1 processing reconstituted in yeast depends on the subunit composition of the m-AAA protease in mitochondria Mol Biol Cell 2007 18 9 3582 3590 17615298 10.1091/mbc.E07-02-0164 PMC1951777

[B58] Jeyaraju DV Sood A Laforce-Lavoie A Pellegrini L Rhomboid proteases in mitochondria and plastids: keeping organelles in shape Biochim Biophys Acta 2013 1833 2 371 380 22634239 10.1016/j.bbamcr.2012.05.019

[B59] Yamaguchi R Perkins G Dynamics of mitochondrial structure during apoptosis and the enigma of Opa1 Biochim Biophys Acta 2009 1787 8 963 972 19245786 10.1016/j.bbabio.2009.02.005 PMC2773558

[B60] Kushnareva YE Gerencser AA Bossy B Ju WK White AD Waggoner J Ellisman MH Perkins G Bossy-Wetzel E Loss of OPA1 disturbs cellular calcium homeostasis and sensitizes for excitotoxicity Cell Death Differ 2013 20 2 353 365 23138851 10.1038/cdd.2012.128 PMC3554330

[B61] Yamaguchi R Lartigue L Perkins G Scott RT Dixit A Kushnareva Y Kuwana T Ellisman MH Newmeyer DD Opa1-mediated cristae opening is Bax/Bak and BH3 dependent, required for apoptosis, and independent of Bak oligomerization Mol Cell 2008 31 4 557 569 18691924 10.1016/j.molcel.2008.07.010 PMC2636708

[B62] White KE Davies VJ Hogan VE Piechota MJ Nichols PP Turnbull DM Votruba M OPA1 deficiency associated with increased autophagy in retinal ganglion cells in a murine model of dominant optic atrophy Invest Ophthalmol Vis Sci 2009 50 6 2567 2571 19234344 10.1167/iovs.08-2913

[B63] Pilsl A Winklhofer KF Parkin, PINK1 and mitochondrial integrity: emerging concepts of mitochondrial dysfunction in Parkinson's disease Acta Neuropathol 2012 123 2 173 188 22057787 10.1007/s00401-011-0902-3

[B64] An HJ Shin H Jo SG Kim YJ Lee JO Paik SG Lee H The survival effect of mitochondrial Higd-1a is associated with suppression of cytochrome C release and prevention of caspase activation Biochim Biophys Acta 2011 1813 12 2088 2098 21856340 10.1016/j.bbamcr.2011.07.017

[B65] An HJ Cho G Lee JO Paik SG Kim YS Lee H Higd-1a interacts with Opa1 and is required for the morphological and functional integrity of mitochondria Proc Natl Acad Sci U S A 2013 110 32 13014 13019 23878241 10.1073/pnas.1307170110 PMC3740888

[B66] Alirol E Martinou JC Mitochondria and cancer: is there a morphological connection? Oncogene 2006 25 34 4706 4716 16892084 10.1038/sj.onc.1209600

[B67] Hoppins S Nunnari J Cell Biology. Mitochondrial dynamics and apoptosis--the ER connection Science 2012 337 6098 1052 1054 22936767 10.1126/science.1224709

[B68] Martinou JC Youle RJ Mitochondria in apoptosis: Bcl-2 family members and mitochondrial dynamics Dev Cell 2011 21 1 92 101 21763611 10.1016/j.devcel.2011.06.017 PMC3156409

[B69] Bender T Martinou JC Where killers meet–permeabilization of the outer mitochondrial membrane during apoptosis Cold Spring Harb Perspect Biol 2013 5 1 a011106 23284044 10.1101/cshperspect.a011106 PMC3579396

[B70] Piquereau J Caffin F Novotova M Prola A Garnier A Mateo P Fortin D le Huynh H Nicolas V Alavi MV Down-regulation of OPA1 alters mouse mitochondrial morphology, PTP function, and cardiac adaptation to pressure overload Cardiovasc Res 2012 94 3 408 417 22406748 10.1093/cvr/cvs117 PMC3863708

[B71] Landes T Emorine LJ Courilleau D Rojo M Belenguer P Arnaune-Pelloquin L The BH3-only Bnip3 binds to the dynamin Opa1 to promote mitochondrial fragmentation and apoptosis by distinct mechanisms EMBO Rep 2010 11 6 459 465 20436456 10.1038/embor.2010.50 PMC2892319

[B72] Quinsay MN Lee Y Rikka S Sayen MR Molkentin JD Gottlieb RA Gustafsson AB Bnip3 mediates permeabilization of mitochondria and release of cytochrome c via a novel mechanism J Mol Cell Cardiol 2010 48 6 1146 1156 20025887 10.1016/j.yjmcc.2009.12.004 PMC2866782

[B73] Herrmann RG Schenk HA, Herrmann RG, Jeon KW, Müller NE, Schwemmler W Eukaryotism, Towards a New Interpretation Eukaryotism and Symbiosis 1997 Berlin Heidelberg: Springer 73 118

[B74] Landes T Martinou JC Mitochondrial outer membrane permeabilization during apoptosis: the role of mitochondrial fission Biochim Biophys Acta 2011 1813 4 540 545 21277336 10.1016/j.bbamcr.2011.01.021

[B75] Hanna RA Quinsay MN Orogo AM Giang K Rikka S Gustafsson AB Microtubule-associated protein 1 light chain 3 (LC3) interacts with Bnip3 protein to selectively remove endoplasmic reticulum and mitochondria via autophagy J Biol Chem 2012 287 23 19094 19104 22505714 10.1074/jbc.M111.322933 PMC3365942

[B76] Zhang H Bosch-Marce M Shimoda LA Tan YS Baek JH Wesley JB Gonzalez FJ Semenza GL Mitochondrial autophagy is an HIF-1-dependent adaptive metabolic response to hypoxia J Biol Chem 2008 283 16 10892 10903 18281291 10.1074/jbc.M800102200 PMC2447655

[B77] Zhang Z Shi R Weng J Xu X Li XM Gao TM Kong J The proapoptotic member of the Bcl-2 family Bcl-2 / E1B-19K-interacting protein 3 is a mediator of caspase-independent neuronal death in excitotoxicity FEBS J 2011 278 1 134 142 21122071 10.1111/j.1742-4658.2010.07939.x

[B78] Nguyen D Alavi MV Kim K-Y Kang T Scott RT Noh YH Lindsey JD Wissinger B Ellisman MH Weinreb RN A new vicious cycle involving glutamate excitotoxicity, oxidative stress and mitochondrial dynamics Cell Death Dis 2011 2 e240 22158479 10.1038/cddis.2011.117 PMC3252734

[B79] Bossy B Petrilli A Klinglmayr E Chen J Lutz-Meindl U Knott AB Masliah E Schwarzenbacher R Bossy-Wetzel E S-Nitrosylation of DRP1 does not affect enzymatic activity and is not specific to Alzheimer's disease J Alzheimers Dis 2010 20 Suppl 2 S513 526 20463395 10.3233/JAD-2010-100552 PMC2893334

[B80] Kanazawa T Zappaterra MD Hasegawa A Wright AP Newman-Smith ED Buttle KF McDonald K Mannella CA van der Bliek AM The C. elegans Opa1 homologue EAT-3 is essential for resistance to free radicals PLoS Genet 2008 4 2 e1000022 18454199 10.1371/journal.pgen.1000022 PMC2265488

[B81] Yarosh W Monserrate J Tong JJ Tse S Le PK Nguyen K Brachmann CB Wallace DC Huang T The molecular mechanisms of OPA1-mediated optic atrophy in Drosophila model and prospects for antioxidant treatment PLoS Genet 2008 4 1 e6 18193945 10.1371/journal.pgen.0040006 PMC2174975

[B82] Tang S Le PK Tse S Wallace DC Huang T Heterozygous mutation of Opa1 in Drosophila shortens lifespan mediated through increased reactive oxygen species production PLoS One 2009 4 2 e4492 19221591 10.1371/journal.pone.0004492 PMC2637430

[B83] Chevrollier A Guillet V Loiseau D Gueguen N de Crescenzo MA Verny C Ferre M Dollfus H Odent S Milea D Hereditary optic neuropathies share a common mitochondrial coupling defect Ann Neurol 2008 63 6 794 798 18496845 10.1002/ana.21385

[B84] Van Bergen NJ Crowston JG Kearns LS Staffieri SE Hewitt AW Cohn AC Mackey DA Trounce IA Mitochondrial oxidative phosphorylation compensation may preserve vision in patients with OPA1-linked autosomal dominant optic atrophy PLoS One 2011 6 6 e21347 21731710 10.1371/journal.pone.0021347 PMC3120866

[B85] Kim JY Hwang JM Ko HS Seong MW Park BJ Park SS Mitochondrial DNA content is decreased in autosomal dominant optic atrophy Neurology 2005 64 6 966 972 15781809 10.1212/01.WNL.0000157282.76715.B1

[B86] Yu-Wai-Man P Sitarz KS Samuels DC Griffiths PG Reeve AK Bindoff LA Horvath R Chinnery PF OPA1 mutations cause cytochrome c oxidase deficiency due to loss of wild-type mtDNA molecules Hum Mol Genet 2010 19 15 3043 3052 20484224 10.1093/hmg/ddq209 PMC2901142

[B87] Chen H Vermulst M Wang YE Chomyn A Prolla TA McCaffery JM Chan DC Mitochondrial fusion is required for mtDNA stability in skeletal muscle and tolerance of mtDNA mutations Cell 2010 141 2 280 289 20403324 10.1016/j.cell.2010.02.026 PMC2876819

[B88] Lodi R Tonon C Valentino ML Manners D Testa C Malucelli E La Morgia C Barboni P Carbonelli M Schimpf S Defective Mitochondrial Adenosine Triphosphate Production in Skeletal Muscle From Patients With Dominant Optic Atrophy Due to OPA1 Mutations Arch Neurol 2011 68 1 67 73 20837821 10.1001/archneurol.2010.228

[B89] Spinazzi M Cazzola S Bortolozzi M Baracca A Loro E Casarin A Solaini G Sgarbi G Casalena G Cenacchi G A novel deletion in the GTPase domain of OPA1 causes defects in mitochondrial morphology and distribution, but not in function Hum Mol Genet 2008 17 21 3291 3302 18678599 10.1093/hmg/ddn225

[B90] Marelli C Amati-Bonneau P Reynier P Layet V Layet A Stevanin G Brissaud E Bonneau D Durr A Brice A Heterozygous OPA1 mutations in Behr syndrome Brain 2011 134 Pt 4 p.e169 author reply e170 10.1093/brain/awq306 21112924

[B91] Alavi MV Fuhrmann N Nguyen HP Yu-Wai-Man P Heiduschka P Chinnery PF Wissinger B Subtle neurological and metabolic abnormalities in an Opa1 mouse model of autosomal dominant optic atrophy Exp Neurol 2009 220 2 404 409 19815013 10.1016/j.expneurol.2009.09.026

[B92] Yu-Wai-Man P Davies VJ Piechota MJ Cree LM Votruba M Chinnery PF Secondary mtDNA defects do not cause optic nerve dysfunction in a mouse model of dominant optic atrophy Invest Ophthalmol Vis Sci 2009 50 10 4561 4566 19443720 10.1167/iovs.09-3634 PMC4034167

[B93] Pidoux G Witczak O Jarnaess E Myrvold L Urlaub H Stokka AJ Kuntziger T Tasken K Optic atrophy 1 is an A-kinase anchoring protein on lipid droplets that mediates adrenergic control of lipolysis EMBO J 2011 30 21 4371 4386 21983901 10.1038/emboj.2011.365 PMC3230380

[B94] Quiros PM Ramsay AJ Sala D Fernandez-Vizarra E Rodriguez F Peinado JR Fernandez-Garcia MS Vega JA Enriquez JA Zorzano A Lopez-Otin C Loss of mitochondrial protease OMA1 alters processing of the GTPase OPA1 and causes obesity and defective thermogenesis in mice EMBO J 2012 31 9 2117 2133 22433842 10.1038/emboj.2012.70 PMC3343468

[B95] Guo Y Chen X Zhang H Li N Yang X Cheng W Zhao K Association of OPA1 polymorphisms with NTG and HTG: a meta-analysis PLoS One 2012 7 8 e42387 22879959 10.1371/journal.pone.0042387 PMC3411762

[B96] Ju WK Kim KY Lindsey JD Angert M Duong-Polk KX Scott RT Kim JJ Kukhmazov I Ellisman MH Perkins GA Weinreb RN Intraocular pressure elevation induces mitochondrial fission and triggers OPA1 release in glaucomatous optic nerve Invest Ophthalmol Vis Sci 2008 49 11 4903 4911 18469184 10.1167/iovs.07-1661 PMC2688012

